# Supplementary figures and images for: map3k1 suppresses terminal differentiation of migratory eye progenitors in planarian regeneration
Source: PLoS Genet. 2025 Mar 17;21(3):e1011457. doi: 10.1371/journal.pgen.1011457 (PMC11981174; doi:10.1371/journal.pgen.1011457)

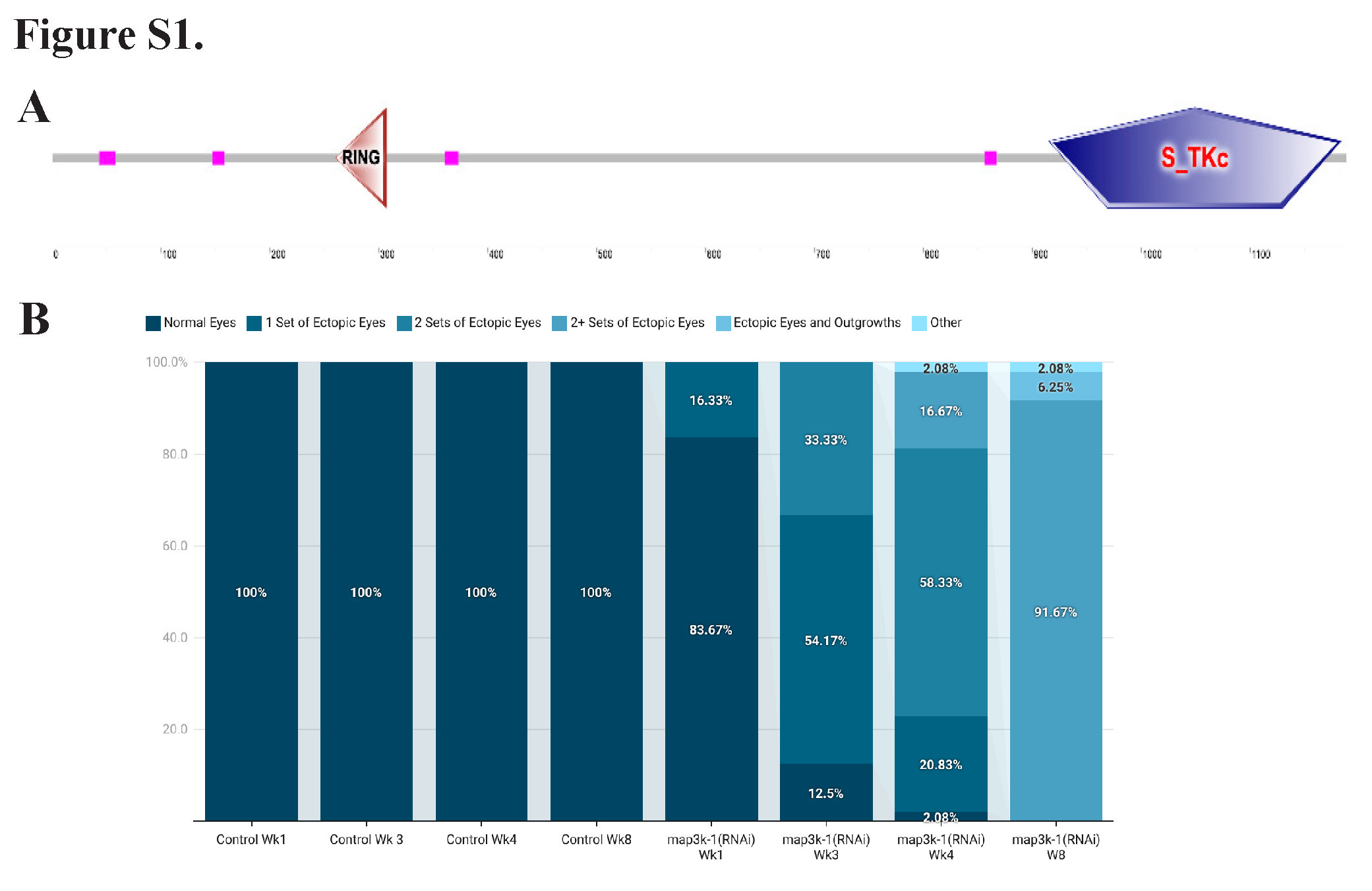

Supplement: S1 Fig — (A) Domain structure of dd_Smed_v6_5198_0_1 (map3k1) containing a RING (E-value=0.0255) domain, a serine/threonine kinase domain (E-value=1.16e-69) characteristic of MAPKs (smart.embl-heidelberg.de). (B) Stacked bar graph quantifying the number of ectopic eyes in control (n=46) versus map3k1(RNAi) (n=48) animals over 8 weeks of RNAi showing that map3k1 inhibition caused ectopic eyes to continue forming over time. (TIF) [file pgen.1011457.s001.tif]

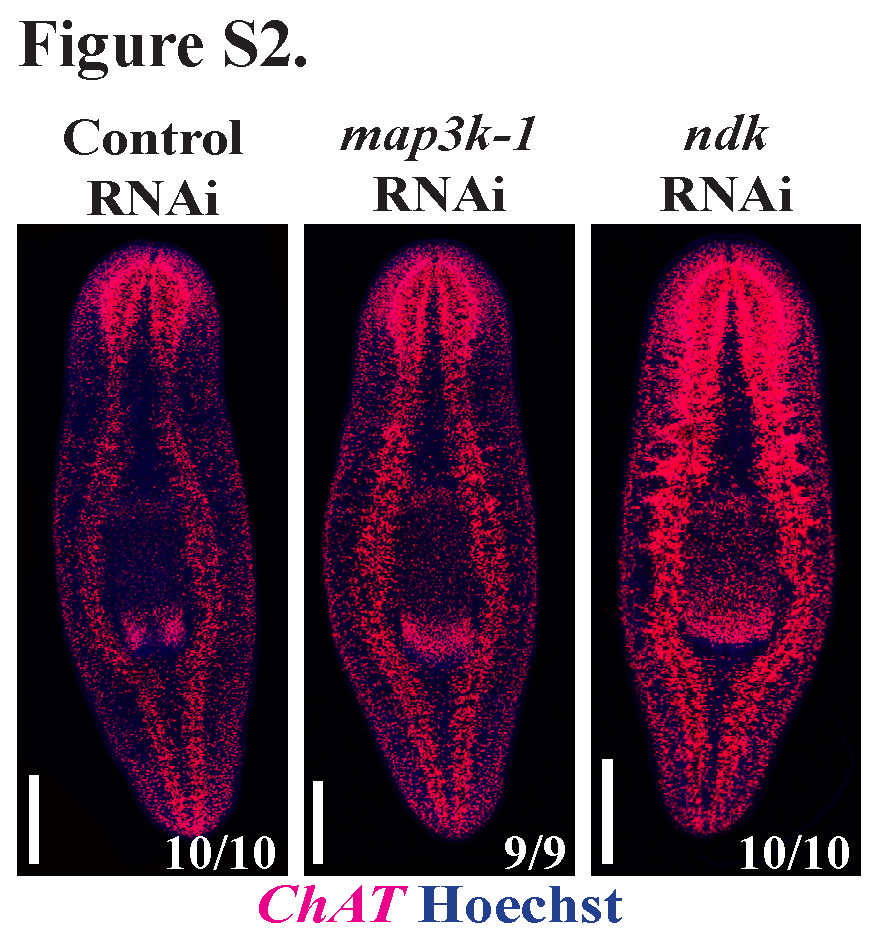

Supplement: S2 Fig — map3k1 RNAi did not increase ChAT+ neuron staining, compared to the ectopic ChAT+ brain branches that formed after ndk RNAi. Scorings indicate how many animals had a ChAT expression pattern that appeared normal (controls and map3k1 RNAi) or had ectopic ChAT+ cells extending laterally from the ventral nerve cords (ndk RNAi). Scale bars, 300μm. (TIF) [file pgen.1011457.s002.tif]

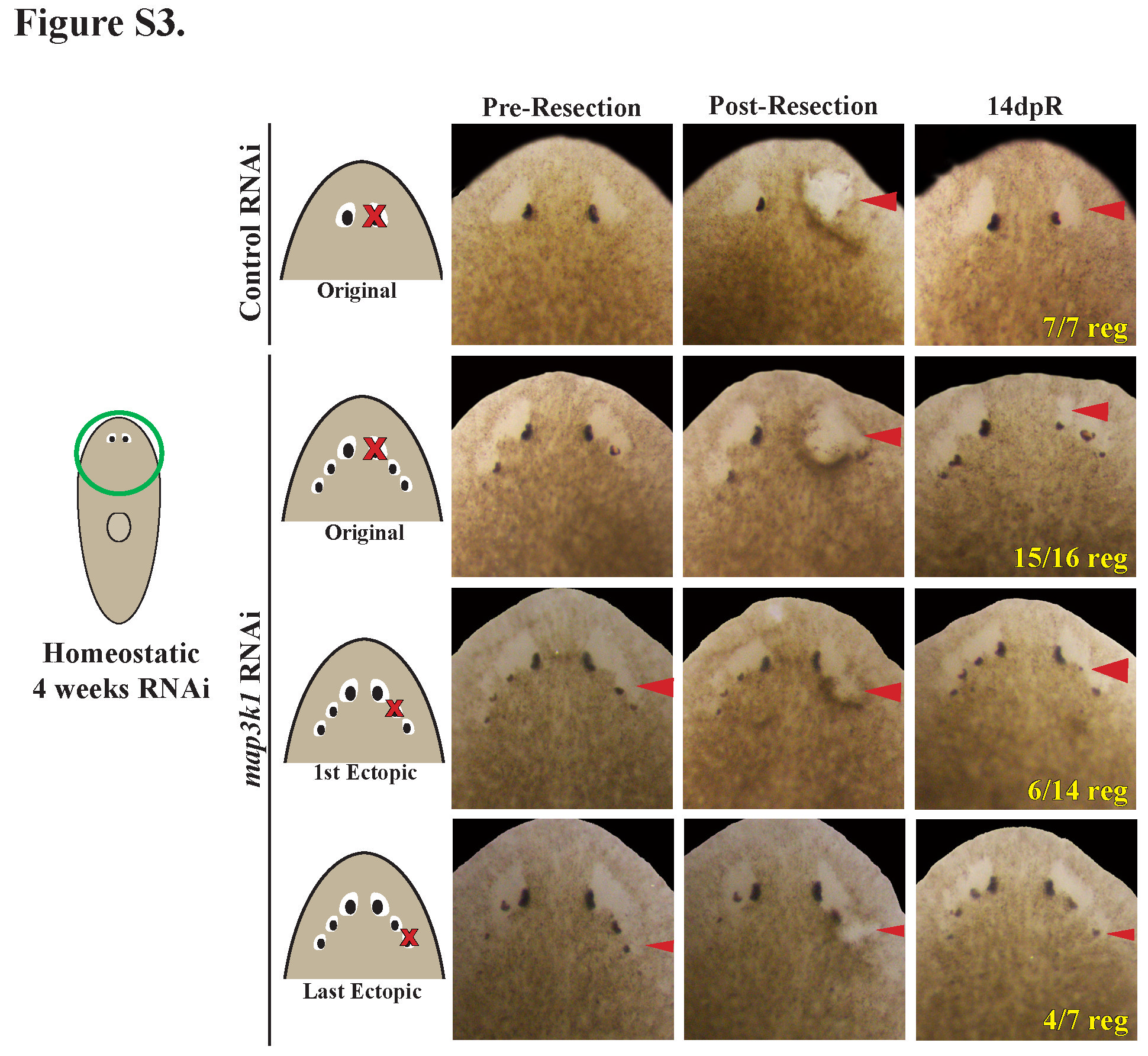

Supplement: S3 Fig — Individual live animals were imaged before, immediately after (post-resection), and 14 days post-surgical eye resection (14dpR) to track whether eye regeneration subsequently occurred. map3k1(RNAi) animals regenerated their original eyes at a high frequency (15/16). Ectopic eyes from these animals were also capable of regeneration, though at lower frequencies. Removal of either the anterior-most ectopic eyes (6/14 eyes regenerated, “1st ectopic”) or the posterior-most ectopic eyes (4/7 eyes regenerated, “last ectopic”) could result in regeneration from the original eye. Sample size, n≥7 animals in each condition. Scorings indicate the number of animals that regenerated an eye in the positions shown. (TIF) [file pgen.1011457.s003.tif]

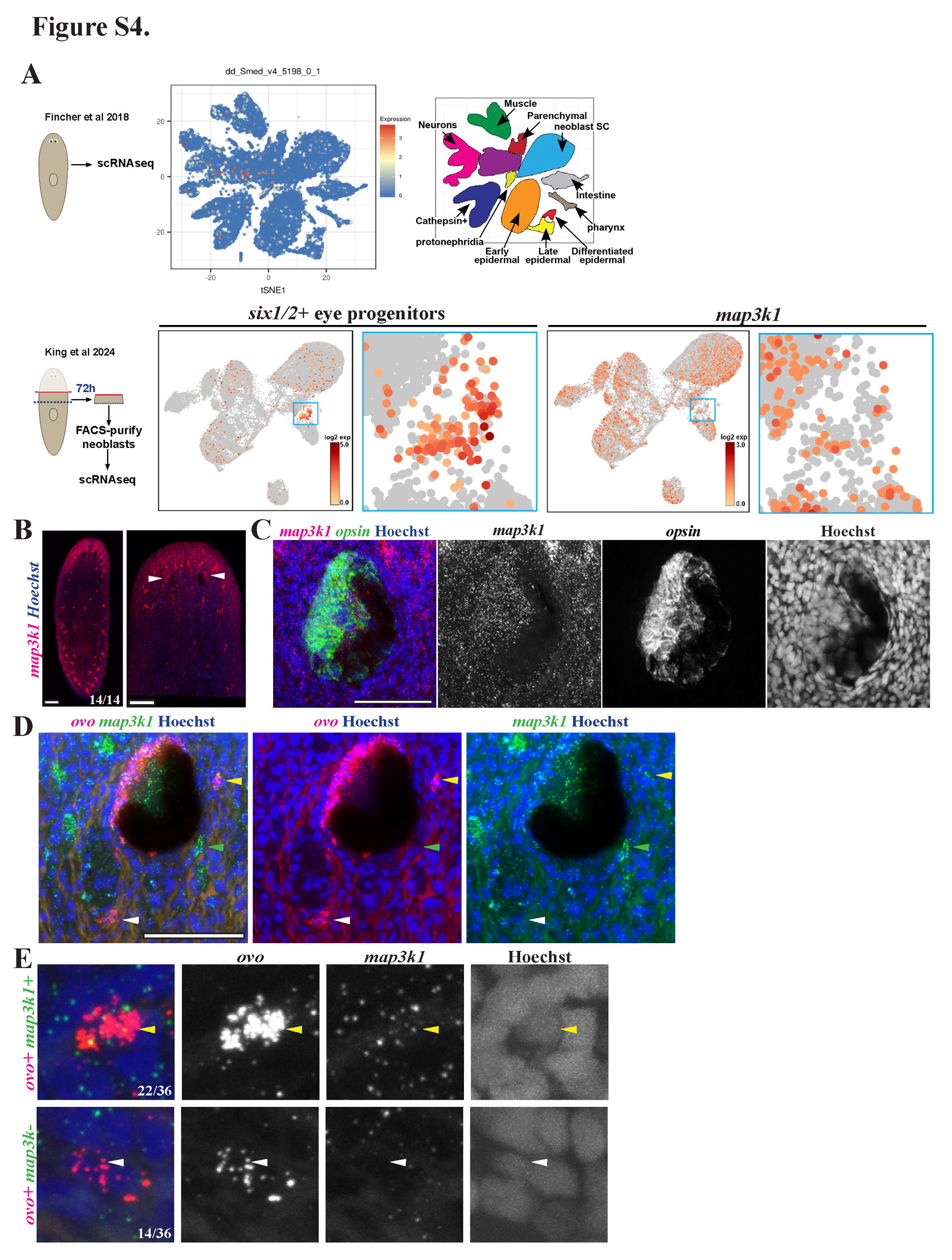

Supplement: S4 Fig — (A) Single-cell RNA sequencing expression profiles of map3k1 expression (A, top panels) in intact animals from Fincher et al. 2018 (36) plotted at digiworm.wi.mit.edu (tSNE plots), and (bottom panels) show map3k1 expression in neoblasts harvested from day 3 anterior-facing blastemas in the process of head and eye regeneration from King et al. 2024 (37) plotted with 10x genomics Loupe (UMAP plots with zoomed insets indicated with blue boxes). map3k1 expression was broad in most tissues in homeostatic animals from the Fincher et al. 2018 (36) cell atlas, including in muscle, neural, and gut clusters. (A, bottom panels) mapk31 positive cells were also present in most clusters of neoblasts isolated from anterior-facing blastemas at 72 hours in the King et al. 2024 (37) early blastema cell atlas, including within rare cells (right panels) located within a cluster of six1/2+ eye progenitors (left panels) identified by that study as produced in early regeneration (37). (B) Maximum projection images of map3k1 expression in homeostatic worms as detected by FISH show map3k1 is expressed broadly throughout the body. Right panel, image showing map3k1 expression in the head and low levels of map3k1 expression in the eyes (arrows). Sample size, n=14 animals. Scale bar, 100μm. (C) Maximum projection images of map3k1 and opsin expression in the eye show some expression of map3k1 in opsin expressing cells (4/4 animals). Scale bar, 50μm. (D-E) Double-FISH detecting ovo and map3k1 expression in homeostatic animals. Panels show either the eye region (D) or higher-magnification view of individual cells (E). Some ovo+ cells expressed low levels of map3k1 (yellow arrowheads) while other ovo+ cells did not have any detectable map3k1 expression (white arrowheads). map3k1 is also broadly expressed, so other unknown map3k1+ovo- cells were identifiable (green arrowhead). Scale bar, 50μm. Panels in (E) show 40X confocal images of ovo and map3k1 expression in homeostatic animals detected through [file pgen.1011457.s004.tif]
